# Oral dabigatran etexilate versus enoxaparin for venous thromboembolism prevention after total hip arthroplasty: pooled analysis of two phase 3 randomized trials

**DOI:** 10.1186/s12959-015-0067-8

**Published:** 2015-11-17

**Authors:** Bengt I. Eriksson, Ola E. Dahl, Nadia Rosencher, Andreas Clemens, Stefan Hantel, Martin Feuring, Jörg Kreuzer, Michael Huo, Richard J. Friedman

**Affiliations:** Department of Orthopaedics, Sahlgrenska University Hospital, SE 413 45 Gothenburg, Sweden; Thrombosis Research Institute, Emmanuel Kaye Building, Manresa Road, London, UK; Innlandet Hospital Trust, Brumunddal, Norway; Department of Anaesthesiology and Intensive Care, Paris Descartes University, Cochin Hospital (AP HP), Paris, France; Corporate Department of Medical Affairs, Boehringer Ingelheim Pharma GmbH & Co. KG, Ingelheim am Rhein, Germany; Center of Thrombosis, University Hospital Mainz, Mainz, Germany; Medical Data Services, Boehringer Ingelheim Pharma GmbH & Co. KG, Ingelheim am Rhein, Germany; Department of Medicine, University of Heidelberg, Heidelberg, Germany; University of Texas Southwestern Medical Center, Dallas, TX USA; Department of Orthopedics, Medical University of South Carolina, Charleston, SC USA

**Keywords:** Arthroplasty, Bleeding, Enoxaparin, Dabigatran, Deep vein thrombosis, Mortality, Prophylaxis, Pulmonary embolism, Venous thromboembolism

## Abstract

**Background:**

Two phase 3 trials compared 28–35 days of treatment with oral dabigatran 220 mg or 150 mg (RE-NOVATE) or 220 mg (RE-NOVATE II) once daily with subcutaneous enoxaparin 40 mg once daily for prevention of venous thromboembolism (VTE) after elective total hip arthroplasty.

**Methods:**

This prespecified pooled analysis compared the outcomes for the dabigatran 220 mg dose with enoxaparin, which included 4,374 patients. Total VTE (venographic and symptomatic) plus all-cause mortality (primary efficacy), major VTE (proximal deep vein thrombosis [DVT] or non-fatal pulmonary embolism) plus VTE-related death, and bleeding events were evaluated. Efficacy analysis was based on the modified intention-to-treat (ITT) population and safety analysis was based on all treated patients. The common risk difference (RD) for dabigatran versus enoxaparin was estimated using a fixed effects model.

**Results:**

Total VTE and all-cause mortality occurred in 6.8 % (114/1,672) and 7.7 % (129/1,682) (RD:–0.8 %, 95 % confidence interval [CI] –2.6 to 0.9) for dabigatran and enoxaparin, respectively. Major VTE plus VTE-related mortality occurred in 2.7 % (46/1,714) and 4.0 % (69/1,711) (RD: –1.4 %, 95 % CI –2.6 to –0.2) of patients receiving dabigatran 220 mg and enoxaparin, respectively. Major bleeding occurred in 1.7 % (37/2,156) and 1.3 % (27/2,157) (RD: 0.5 %, 95 % CI –0.2 to 1.2), for dabigatran and enoxaparin respectively.

**Conclusions:**

Extended prophylaxis with oral dabigatran 220 mg once daily was as effective as enoxaparin 40 mg once daily in reducing the risk of total VTE and all-cause mortality after total hip arthroplasty, with a similar bleeding profile. The clinically relevant outcome of major VTE and VTE-related death was significantly reduced with dabigatran versus enoxaparin.

**Trial registration:**

NCT00657150 and NCT00168818

## Background

Dabigatran etexilate (hereafter referred to as dabigatran) is an orally administered direct, reversible thrombin inhibitor for the prevention and treatment of various thromboembolic disorders. Two previously reported phase 3 trials (RE-NOVATE and RE-NOVATE II) [1, 2] compared the efficacy and safety of dabigatran (220 mg or 150 mg once daily, started 1–4 h after surgery) with enoxaparin (40 mg once daily, started at least 12 h before surgery) for the prevention of venous thromboembolism (VTE) and all-cause mortality after elective total hip arthroplasty. In both of these studies, the non-inferiority of dabigatran 220 mg over enoxaparin 40 mg for the primary efficacy endpoint, total VTE (the composite of symptomatic and asymptomatic venographic deep vein thrombosis [DVT], non-fatal pulmonary embolism [PE]) plus all-cause mortality, was demonstrated. Bleeding and adverse event (AE) rates with dabigatran were low and similar to those reported for enoxaparin. Dabigatran 220 mg once daily (starting with a half dose 1–4 h after the end of surgery) is now approved in more than 100 countries for thromboprophylaxis in patients undergoing total hip arthroplasty.

The prespecified pooled analysis of these two studies was planned to compare the effect of dabigatran 220 mg and enoxaparin 40 mg once daily on the primary efficacy endpoint of total VTE and all-cause mortality in patients undergoing total hip arthroplasty.

## Methods

### Study design and setting

RE-NOVATE and RE-NOVATE II were prospective, double-blind, double-dummy, randomized, multicentre, non-inferiority studies.

Participants were adults aged at least 18 years who were scheduled for primary elective total hip arthroplasty. The two trials had identical study eligibility criteria and were designed to be as similar as possible. Briefly, patients were randomized to treatment with oral dabigatran 220 mg or 150 mg once daily (the latter dose was not used in RE-NOVATE II), or subcutaneous enoxaparin 40 mg once daily started the evening before surgery; in some countries enoxaparin treatment was started postoperatively in accordance with local practice.

The first dose of dabigatran was halved and given 1–4 h after wound closure, provided clinical assessment of perioperative and postoperative bleeding and drainage indicated adequate hemostasis. If administration was delayed until the day after surgery, a full dose was given, followed by a second dose at least 12 h later. Treatment was continued until mandatory bilateral venography at 28–35 days. In both trials, the treatment period was defined as the time from first dose to 3 days after the last dose. Continued VTE prophylaxis was at the discretion of the treating physician. Patients attended a clinical follow-up visit 3 months after surgery. Concomitant administration of low dose aspirin (<160 mg) and selective cycloxygenase-2 inhibitors was allowed during treatment. Elastic compression stockings were permitted, but intermittent pneumatic compression devices were prohibited.

Both studies were approved by National Independent Ethics Committees and conducted according to the Declaration of Helsinki (October 1996 version). All patients gave signed informed consent prior to entry.

### Outcome measures

The prespecified primary efficacy endpoint of this pooled analysis was identical with that of the individual trials, i.e., the composite of total VTE and all-cause mortality. The endpoint was analyzed in the modified intention-to-treat (mITT) population, comprising all randomized and treated patients who underwent elective total hip arthroplasty and had evaluable adjudicated data on VTE (venographic confirmation in both legs or symptomatic event) or died during the treatment period. The main secondary efficacy outcome was the composite of major VTE (venographic or symptomatic proximal DVT and/or PE) and VTE-related mortality during treatment. Additional predefined secondary efficacy outcomes during the treatment period included total DVT (venographic or symptomatic), proximal DVT (venographic or symptomatic), and symptomatic DVT and/or PE.

All efficacy endpoints were based on assessments made by the same blinded Independent Venous Thromboembolic Event Adjudication Committee. Mandatory bilateral venography was performed within 24 h of the last oral dose, as described previously [3]. Suspected symptomatic DVT during treatment or follow-up was confirmed by ultrasound or venography. Symptoms suggestive of PE mandated confirmation by ventilation-perfusion scintigraphy, pulmonary angiography or spiral chest computer tomography, depending on local center preference. Deaths were considered related to VTE if they were categorized as “VTE related” or “unexplained" by the Independent Adjudication Committee.

All randomized patients who received at least one dose of study treatment were evaluable for safety. The main safety endpoint was the frequency of major bleeding events (which, different to previously performed studies, also included bleeding from the surgery wound site) occurring between intake of the first dose of study medication and 3 days after the last dose. Secondary safety outcomes included the composite of major and clinically relevant non-major bleeding events, any bleeding events during treatment, liver enzyme elevations (≥3 x the upper limit of the normal reference range (ULN) for serum alanine aminotransferase [ALT]) and acute coronary events (defined as confirmed unstable angina, myocardial infarction [MI], and cardiac death). This is in line with a number of pooled analyses from total knee and hip replacement trials with dabigatran, which included more than 8,000 patients [4, 5]. Major, clinically relevant, non-major and minor bleeding events were defined according to accepted guidelines [6], as reported previously [3]. In particular, the definition of major bleeding includes wound site bleeding events, in accordance with recommended guidelines [6]. ALT elevations ≥3 x ULN and any suspected acute coronary syndrome events were reviewed by an Independent Committee who were blinded to treatment allocation. An assessment of causality was provided for each of the reviewed patient cases.

### Statistical analysis

The statistical analysis plan described here was planned before commencement of the RE-NOVATE II trial in accordance with regulatory recommendations [7].

For each trial, the difference in the proportion of patients with an event (efficacy or safety) was compared between dabigatran 220 mg and enoxaparin 40 mg as a risk difference (RD) as this was considered the most clinically meaningful measure. A common RD estimate across the two trials was calculated using a fixed-effects model (maximum likelihood estimation) [8], based on inverse variance weights for combined results from the individual trials, and compared with results obtained with a random effects model (DerSimonian and Laird method). The RD was not expected to differ between the studies. Heterogeneity of the common RD between the studies was assessed using Cochran’s χ2 and the I^2^ statistic; p <0.10 was considered to denote statistically significant heterogeneity and where I^2^ was greater than 50 %, heterogeneity was considered substantial [9]. Pooled event rate data for each treatment group are presented alongside the common RD results obtained from the pooled analysis.

Subgroup analyses were planned to investigate the influence of age, body weight, gender, and renal function (determined from calculated baseline creatinine clearance) on the incidence of the primary efficacy outcome, as well as bleeding events. For these comparisons the odds ratio and 95 % confidence intervals (CIs) for the analyzed subgroups were calculated using a fixed-effects model for the efficacy and safety endpoints.

Sensitivity analyses were conducted to explore the robustness of the results. The impact of missing or non-evaluable venography data, based on imputation of missing values using best and worst case scenarios (all treatment success or all treatment failure), was investigated to ensure that missing data did not affect the power of the trial or bias any estimation of the treatment effect.

## Results

### Study population

**Fig. 1:**
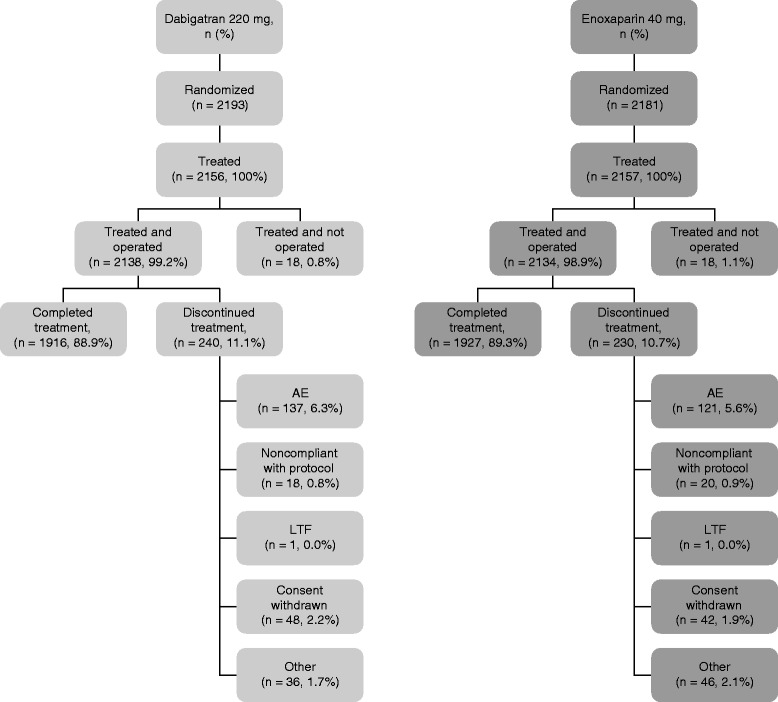
Flow of patients through the study

A total of 4,374 patients were randomized across 20 countries between December 2004 and May 2009, of whom 4,313 were treated and therefore evaluable for safety. 4,272 patients were operated upon and treated with oral dabigatran 220 mg (*n* = 2,138) or subcutaneous enoxaparin 40 mg (*n* = 2,134) (Fig. 1). A further 918 (21.5 %) patients were excluded from the mITT population, as usual in all studies with venography, mainly because bilateral venography was not performed (usually declined by the patient) or the venograms were considered indeterminate by the venography adjudication committee. This percentage is consistent with that reported in contemporary studies using venography as an endpoint [3, 10, 11]. In total, 240 (11.2 %) patients allocated dabigatran and 230 (10.7 %) allocated enoxaparin discontinued treatment. The primary reasons for discontinuation were similar between groups. The two groups were well balanced in terms of demographic and surgical characteristics (Table 1).

**Table 1:** Demographic and baseline characteristics

	Dabigatran 220 mg	Enoxaparin 40 mg
	(*n* = 2,156)	(*n* = 2,157)
Number of treated patients	2,156	2,157
Age, yr	63 ± 11	63 ± 11
Females, *n* (%)	1177 (54.6)	1152 (53.4)
Weight, kg	79 ± 16	79 ± 16
Body mass index^a^	27.8 ± 4.7	27.6 ± 4.6
Previous VTE, *n* (%)	65 (3.0)	54 (2.5)
Creatinine clearance^b^, mL/min	92 ± 31	93 ± 31
Race^c^, *n* (%)		
White	2,051 (95.1)	2,052 (95.1)
Asian	96 (4.4)	88 (4.1)
Black	5 (0.2)	12 (0.6)
Other	4 (0.2)	5 (0.2)
Geographical region, *n* (%)		
Western Europe	1,282 (59.5)	1,291 (59.9)
Central Europe	474 (22.0)	470 (21.8)
North America	170 (7.9)	168 (7.8)
India	91 (9.0)	88 (4.1)
Australia/New Zealand/South Africa	139 (6.4)	140 (6.5)
Patients treated and operated, *n* (%)	2,138 (99.2)	2,134 (98.9)
Anesthesia^d^, *n* (%)		
General alone	525 (24.4)	503 (22.3)
Neuraxial alone^e^	1,461 (67.8)	1,486 (65.8)
Combination^f^	149 (6.9)	144 (6.4)
Mean duration of surgery ± SD, min	85.4 ± 30.2	85. ±30.4
Study treatment		
Mean time to first subcutaneous injection^g,h^ in relation to surgery, hr	–15.6 ± 20.7	–15.2 ± 13.5
Mean time to first oral dose postsurgery^h^, hr	3.1 ± 2.6	3.2 ± 2.7^g^
Median (range) duration of hospital stay^i^, d	8.5 (3–51)	8.5 (3–26)
Median (range) treatment duration, d	31.6 (1–89)	31.7 (1–49)

Data are given as mean ± SD except where indicated

*SD* standard deviation, *VTE* Venous thromboembolism

^a^Body mass index was defined as weight in kilograms divided by square of height in meters; ^b^Creatinine clearance rates were calculated using the Cockcroft–Gault formula; ^c^As reported by the investigator; ^d^Patients may have had more than one type of anesthetic; ^e^Includes spinal and epidural anesthesia; ^f^Peripheral nerve block plus general or neuraxial anesthesia; ^g^26 patients in RE-NOVATE II group received their first dose postsurgery; ^h^Includes both active treatment and placebo; ^i^Time from surgery until day of discharge, data available in RE-NOVATE for 1,136 and 1,140 patients, respectively

### Efficacy outcomes

Efficacy outcomes are summarized in Table 2. The primary outcome (the composite of total VTE and all-cause mortality) occurred in 114 (6.8 %) patients treated with dabigatran 220 mg and 129 (7.7 %) treated with enoxaparin 40 mg (RD: –0.8 %, 95 % CI –2.6 to 0.9; *p* = 0.35). Distal (below knee) DVT detected by venography was the most frequent component of the primary endpoint. There were four deaths during treatment; three in the dabigatran group (one related to VTE), and one in the enoxaparin group not related to VTE.

**Table 2:** Efficacy outcomes, modified ITT population. Data are given as n/N (%)

Outcome	Dabigatran 220 mg	Enoxaparin 40 mg	Risk difference vs. enoxaparin, % (95 % CI)^a^	p value
Total VTE and all-cause mortality				
Pooled data	114/1,672 (6.8)	129/1,683 (7.7)	–0.8 (–2.6, 0.9)	0.35
RE-NOVATE	53/880 (6.0)	60/897 (6.7)	–0.7 (–2.9, 1.6)	
RE-NOVATE II	61/792 (7.7)	69/786 (8.8)	–1.1 (–3.8, 1.6)	
Major VTE^b^ and VTE-related mortality^c^				
Pooled data	46/1,714 (2.7)	69/1,712 (4.0)	–1.4 (–2.6, –0.2)	0.03
RE-NOVATE	28/909 (3.1)	36/917 (3.9)	–0.8 (–2.5, 0.8)	
RE-NOVATE II	18/805 (2.2)	33/795 (4.2)	–1.9 (–3.6, –0.2)	
Symptomatic events				
Symptomatic VTE^d^	17/2,138 (0.8)	16/2,134 (0.7)		
Symptomatic DVT	6/2,138 (0.3)	5/2,134 (0.2)		1.00
Symptomatic PE	6/2,138 (0.3)	5/2,134 (0.2)		1.00
Death	3/2,138 (0.1)	1/2,134 (0.0)		0.62
Total asymptomatic DVT	100/1,665 (6.0)	122/1,677 (7.3)		
Proximal	35/1,709 (2.0)	63/1,706 (3.7)		
Distal only	65/1,666 (3.9)	59/1,679 (3.5)		
Total study period (treatment + follow-up)				
Symptomatic VTE + all-cause mortality	18/2,048 (0.9)	19/2,059 (0.9)		0.09

N = number of patients included within each population with percentage in parentheses

*CI* confidence interval, *DVT* deep vein thrombosis, *ITT* intention-to-treat, *PE* pulmonary embolism, *VTE* venous thromboembolism

^a^Based on normal approximation of binomial distribution for single trial and the fixed effects approach using a weighted average (inverse variance) for pooled analyses; ^b^Major VTE was defined as venographic and symptomatic proximal DVT and/or non-fatal PE; ^c^VTE-related mortality included fatal PE and deaths where VTE cannot be excluded; ^d^Includes any symptomatic DVT (proximal or distal) and non-fatal or fatal symptomatic PE in patients in the safety population who had undergone surgery

The main secondary outcome (composite of major VTE and VTE-related mortality) occurred in 2.7 % with dabigatran versus 4.0 % with enoxaparin (RD: –1.4 %, 95 % CI –2.6 to –0.2; *p* = 0.03). Symptomatic DVT occurred in 0.3 % versus 0.2 % (*p* = 1.00), respectively. Over the whole 3-month (treatment plus follow-up) study period the rate of symptomatic VTE plus all-cause mortality was 0.9 % in each treatment group.

There was a significant difference in the risk for proximal DVT (venographic or symptomatic) for dabigatran versus enoxaparin (RD: –1.4 %, 95 % CI –2.6 to –0.3; *p* = 0.02). No difference in total DVT (venographic or symptomatic) (RD: –1.1 %, 95 % CI –2.7 to 0.6; *p* = 0.22) was observed. The incidence of symptomatic DVT and PE during treatment was comparable across treatments (*p* = 1.0) (Table 2). Similar results were reported for the main secondary outcome.

Bleeding-related outcomes are summarized in Table 3. There was no difference in major bleeding rates between the two groups; 37 (1.7 %) with dabigatran versus 27 (1.3 %) with enoxaparin (RD: 0.5 %, 95 % CI –0.2 to 1.2; *p* = 0.19). Only one fatal bleeding event in the dabigatran group was reported. Of the 37 major bleeding events with dabigatran, 19 (51.4 %) occurred before any active study drug had been administered. In contrast, all bleeding events in the enoxaparin group occurred after the first dose, which was given preoperatively in 94 % of patients. Similarly, there was no difference between the groups in the rate of major or non-major clinically relevant bleeding events (5.0 % versus 4.0 % dabigatran and enoxaparin, respectively, *p* = 0.13), overall bleeding rates or the requirement for blood transfusion.

**Table 3:** Bleeding-related outcomes, safety population

Outcome	Dabigatran 220 mg	Enoxaparin	Risk difference vs. enoxaparin, %	*p* value
	(*n* = 2,156)	(*n* = 2,157)	(95 % CI)	
Bleeding Events				
Major, Total no. patients, % (95 % CI)^a^	37 (1.7, 1.2-2.4 %)	27 (1.3, 0.8-1.8 %)	0.5 % (–0.2, 1.2)	0.20
Fatal	1 (0.05)	0 (0)		
In a critical organ	2 (0.1)	0 (0)		
Clinically overt associated with 20 g/L or more fall in hemoglobin	31 (1.4)	19 (0.9)		
Clinically overt leading to transfusion of two or more units of packed cells or whole blood	33 (1.5)	22 (1.0)		
Warranting treatment cessation	1 (0.05)	1 (0.05)		
Leading to re-operation	2 (0.1)	3 (0.1)		
Onset of events – No. events/total no. patients (%)				
-Before the first oral dose	19/37 (51.4)	10/28 (35.7)		
-After the first oral dose	18/37 (48.6)	18/28 (64.3)		
Clinically relevant non-major bleeding	71 (3.3)	60 (2.8)		
Major or clinically relevant non-major bleeding	108 (5.0)	87 (4.0)	1.0 % (–0.3, 2.2)	0.13
Minor bleeding	131 (6.1)	128 (5.9)		
Any bleeding events	239 (11.1)	215 (10.0)		
Patients receiving blood transfusions - n/N (%)	858/2,138 (40.1)	880/2,134 (41.2)		

Data are given as number (%) of patients except where indicated

*CI* confidence interval

^a^Patients may have been included in more than one category

### Subgroup analyses

**Fig. 2:**
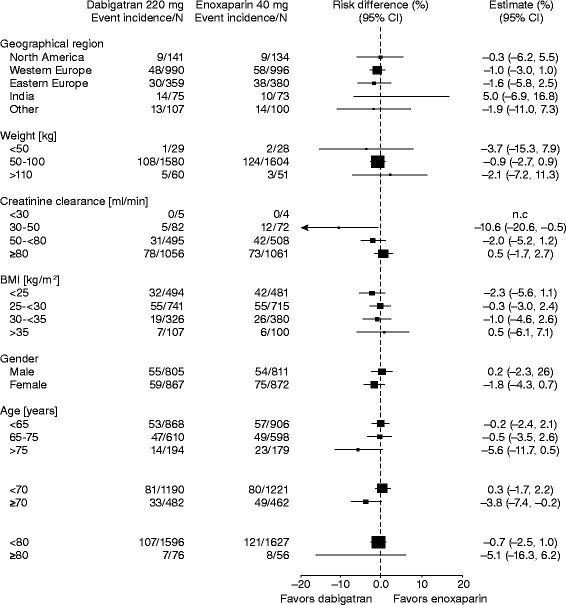
Total VTE and all-cause mortality during treatment period by subgroup (risk differences)

Treatment with dabigatran resulted in consistent reductions in the primary outcome irrespective of age (<65, 65–75, >75 years, *p* = 0.05), weight (≤70, >70–90, >90 kg) (*p* = 0.50), gender (*p* = 0.54) or renal function (calculated creatinine clearance >80, 50–80, <50 mL/min at baseline, *p* = 0.32). There were no differences in the primary outcome across additional subgroups according to treatment (Fig. 2).

The results remained robust in sensitivity analyses (data not shown), indicating that missing venography data did not bias any estimation of the treatment effect. There were no differences between results obtained using the fixed versus random effects model.

### Adverse events

The AE profile of each treatment was similar (Table 4). Postoperative wound infection was reported as an AE in 37 patients, 16 (0.7 %) in the dabigatran group and 21 (1.0 %) in the enoxaparin group. AEs leading to treatment discontinuation occurred in 6.2 % and 5.5 % of the dabigatran and enoxaparin groups, respectively (Table 4). Less than 1 % of patients in either group had an adjudicated MI or ischemic stroke during or after treatment. Fifteen patients (8 [0.4 %] in the dabigatran group and 7 [0.3 %] in the enoxaparin group) had cardiovascular events (defined as ischemic stroke or MI) after >3 days off study drug.

**Table 4:** Adverse events, safety population

Outcome	Dabigatran 220 mg	Enoxaparin 40 mg
	(*n* = 2,156), *n* (%)	(*n* = 2,157), *n* (%)
AEs during treatment		
Total with AEs	1,563 (72.5)	1,588 (73.6)
Serious AEs	146 (6.8)	141 (6.5)
AEs leading to treatment discontinuation	134 (6.2)	118 (5.5)
Drug-related AEs (investigator evaluation)	191 (8.9)	199 (9.2)
Wound infections^a^	16 (0.7)	21 (1.0)
Cardiovascular events		
Myocardial infarction	2 (<0.1)^b^	6 (0.3)
Ischemic stroke	0	1 (0.1)
ALT elevation; no. (%) patients		
>3 x ULN anytime post baseline	71/2,101 (3.4)^c^	115/2,097 (5.5)^c^
>3 x ULN plus bilirubin >2 x ULN during treatment period	3/2,092 (0.1)^d^	0/2,096

Data are given as number (%) of patients except where indicated

*AE* adverse event, *ALT* alanine aminotransferase, *ULN* upper limit of normal

^a^Includes wound hematomas, wound secretions, wound drainage and wound hemorrhage; ^b^A further event in RE-NOVATE II occurred in the follow-up period; ^c^Number of patients with abnormality/total number of patients having tests; ^d^A further patient in RE-NOVATE II had an elevation 3 months postsurgery while still in the follow-up period. None of the patients met the criteria for severe drug-induced hepatotoxicity [12]. One of these patients was diagnosed with acute cholangitis but a definitive diagnosis was not made in the other patient

Moderate liver enzyme elevation (ALT levels >3 x ULN) at any time after baseline occurred in 3.4 % of the dabigatran group and 5.5 % of the enoxaparin group (Table 4). In three patients in the dabigatran group, there was an associated two-fold increase in bilirubin elevation. None of these cases met the criteria (Hy’s law) for severe drug-induced hepatotoxicity [12].

## Discussion

The prespecified pooled analysis of these two studies was planned to compare the effect of dabigatran 220 mg and enoxaparin 40 mg once daily on the primary efficacy endpoint of total VTE and all-cause mortality in patients undergoing total hip arthroplasty. This pooled analysis of data from ~4,300 patients undergoing elective total hip arthroplasty in the two RE-NOVATE studies adds to the evidence base for dabigatran for prevention of thromboembolic complications.

Dabigatran 220 mg was as effective as enoxaparin 40 mg in decreasing the risk of VTE and all-cause mortality at 5 weeks. These results were consistent across age, weight, gender, or creatinine clearance subgroups. It is notable that the rate of major VTE and VTE-related death was significantly lower with dabigatran (*p* = 0.03), with a 1.4 % absolute reduction in risk versus enoxaparin. This rate compares with the absolute reduction (0.7 to 1.7 %) observed in studies with the oral FXa inhibitors (apixaban and rivaroxaban) versus enoxaparin in patients undergoing hip arthroplasty and receiving prophylaxis for an equivalent extended duration [10, 11]. Thus, dabigatran may have similar benefits in reducing more clinically relevant, proximally located lower limb thrombi as other available oral anticoagulants.

The risk of bleeding was similar to that for enoxaparin. Bleeding rates (major, clinically relevant non-major, and minor bleeding) did not differ statistically between dabigatran and enoxaparin across the two studies. All but 33 % of the major bleeding events were reported to have occurred after day 3, although the onset of bleeding could have started earlier.

Taken together, these results indicate that dabigatran is a useful prophylactic therapy in this clinical setting. By pooling the data from the two studies, a higher grade of power and prediction was achieved and allowed for an improved understanding. Specifically, the rarer, but clinically more relevant outcomes (major VTE and VTE-related mortality, or proximal VTE) regarding the performance of the oral anticoagulant dabigatran in comparison to subcutaneous enoxaparin could be investigated. Sensitivity analysis also demonstrated that missing data did not affect the power of the trial or bias any estimation of the treatment effect.

Other important outcomes included treatment-related wound complications and postoperative wound infections, all of which occurred with a similar frequency in each group. Such complications are clinically relevant, since bleeding assessment and wound-related AEs can affect surgical outcome and influence decisions regarding the provision of extended out-of-hospital prophylaxis. The AE profile, including liver enzyme elevations and cardiovascular events, was similar between dabigatran and enoxaparin.

Dabigatran is administered orally and is therefore more convenient than parenterally administered anticoagulants such as enoxaparin, particularly for out-of hospital prophylaxis. This is of relevance when considering current practice in the use of VTE prophylaxis. Despite guidelines recommending use of prophylaxis for up to 35 days after hip arthroplasty [13], compliance may be less than ideal. In one report, only ~75 % of patients treated with parenteral low-molecular weight heparin (e.g., enoxaparin) in hospital continued to receive it after discharge [14]. It is anticipated that these practical advantages are likely to confer cost advantages, supported by recent analysis. Indeed, economic evaluation, from the perspective of the UK National Health System, showed that extended prophylaxis with dabigatran was cost-effective compared with enoxaparin, given the substantial reduction in costs due to oral administration (pre- and postdischarge from hospital) [15]. Similar savings have been reported in other country-specific analyses from the healthcare system perspective [16, 17].

## Conclusions

In conclusion, this pooled analysis of two trials comparing dabigatran with enoxaparin demonstrated that dabigatran was comparable to enoxaparin in preventing total VTE and all-cause mortality when used for the same duration, with a similar risk of bleeding and AEs. In clinical practice, dabigatran can be considered an attractive thromboprophylaxis in patients undergoing elective hip arthroplasty, with potential economic advantages.

## Abbreviations

ALT: alanine aminotransferase
CI: confidence interval
DVT: deep vein thrombosis
ITT: intention-to-treat
MI: myocardial infarction
mITT: modified intention-to-treat
PE: pulmonary embolism
RD: risk difference
ULN: upper limit of the normal reference range
VTE: venous thromboembolism
